# ECMO after cardiac surgery: a single center study on survival and optimizing outcomes

**DOI:** 10.1186/s13019-021-01638-0

**Published:** 2021-09-19

**Authors:** Jennifer M. Brewer, Anthony Tran, Jielin Yu, M. Irfan Ali, Constantine M. Poulos, Jonathan Gates, Jason Gluck, David Underhill

**Affiliations:** 1 Department of General Surgery, University of Connecticut, 263 Farmington Ave, Farmington, CT 06030 USA; 2 Division of Trauma and Acute Care Surgery, Hartford Healthcare, Hartford, USA; 3 Department of Cardiology and Mechanical Circulatory Support, Hartford Healthcare, Hartford, USA; 4 Division of Cardiac Surgery, Hartford Healthcare, Hartford, USA

**Keywords:** Cardio-thoracic surgery, ECMO, Cardiac transplantation, Critical care, LVAD, Cardiac surgery

## Abstract

**Background:**

The study purpose is to examine survival prognostic and extracorporeal membrane oxygenation (ECMO) application outcomes at our tertiary care center.

**Methods:**

This is a retrospective analysis, January 2014 to September 2019. We analyzed 60 patients who underwent cardiac surgery and required peri-operative ECMO. All inpatients with demographic and intervention data was examined. 52 patients (86.6%) had refractory cardiogenic shock, 7 patients (11.6%) had pulmonary insufficiency, and 1 patient (1.6%) had hemorrhagic shock, all patients required either venous-arterial (VA) (n = 53, 88.3%), venous-venous (VV) (n = 5, 8.3%) or venous-arterial-venous (VAV) (n = 2, 3.3%) ECMO for hemodynamic support. ECMO parameters were analyzed and common postoperative complications were examined in the setting of survival with comorbidities.

**Results:**

In-hospital mortality was 60.7% (n = 37). Patients who survived were younger (52 ± 3.3 vs 66 ± 1.5, p < 0.001) with longer hospital stays (35 ± 4.0 vs 20 ± 1.5, p < 0.03). Survivors required fewer blood products (13 ± 2.3 vs 25 ± 2.3, p = 0.02) with a net negative fluid balance (− 3.5 ± 1.6 vs 3.4 ± 1.6, p = 0.01). Cardiac re-operations worsened survival.

**Conclusion:**

ECMO is a viable rescue strategy for cardiac surgery patients with a 40% survival to discharge rate. Careful attention to volume management and blood transfusion are important markers for potential survival.

## Background

Myocardial dysfunction after cardiac surgical intervention occurs in about 3–8% of patients [1, 2]. Patients are typically separated from cardiopulmonary bypass with inotropes/vasopressors or intra-aortic balloon counterpulsation [3–5]. In the event of refractory cardiac and/or pulmonary dysfunction additional mechanical circulatory support may be required. Extracorporeal membrane oxygenation (ECMO) is a bridging mechanical circulatory support with promising results seen in a multitude of post cardiotomy procedures with poor residual cardiac function [1, 6–9]. Prognostic factors previously identified include: preventing left ventricular overloading, pulmonary edema, lung injury, and myocardial damage. Additional prognostic indicators are low oxygen pressure and low oxygen saturation of ECMO tubing, advanced age, pre-operative co-morbidities, the type of surgical procedure, and high blood product requirement [10–17]. We analyzed 60 patients who underwent cardiac surgery and required peri-operative ECMO support from January 2014 to September 2019. The purpose of this study is to delineate prognostic factors of survival, discuss the outcomes, and elaborate on the application of ECMO at a tertiary care center.

## Methods

From January 2014 to September 2019, we performed over 6000 cardiac surgeries and over 250 ECMO implants of which 60 were placed in cardiotomy patients. The patients requiring ECMO support following primary cardiac surgery underwent the following procedures: coronary artery bypass grafting (CABG), valvular surgery, CABG with concomitant valvular surgery, heart transplantation, aortic root repair, left ventricular assistant device (LVAD) implantation, descending aortic repair, and other procedures. Nine cases were implanted at institutions outside our network and brought back to our hub hospital. Patients were placed on venous-arterial (VA), venous-venous (VV), or veno-arterio-venous (VAV) ECMO. Indications for placing a patient on ECMO included cardiogenic shock, pulmonary insufficiency or hemorrhagic shock. Cardiogenic shock was defined as more than two vasoactive drugs and escalating care, or intra-aortic balloon pump (IABP) with more than one vasoactive drug and escalating care, or cardiac arrest with return of spontaneous circulation. Respiratory failure was defined as pO2 < 50 and FiO2 > 60% for at least 12 h, or pH < 7.2 despite optimal vent settings, or P/F ratio < 80 with high levels of PEEP. We used regional SO^2^ monitors to ensure that when the femoral artery was cannulated there was perfusion to that extremity from our routinely performed jump grafts. We have a specific ECMO anticoagulation protocol for post cardiac surgery patients. Heparin was reversed in the operating room (OR) with protamine sulfate and anticoagulation was typically held for 24 h post-operatively. Patients were then usually anticoagulated with 500-700units/per hour of heparin as a fixed dose and without a bolus for 6 h then gently uptitrated to achieve an anti-Xa level of 0.3–0.5. If cannulation occurred in the OR we performed direct surgical cannulation of the right atrium and aorta. The size of the cannula was surgeon preference. Usually for the atrium this is about a 28–32 French cannula, for the aorta this is about a 20 French cannula. We used multilevel measures to ensure that the cannulas are secured, such as pledgeted horizontal mattress sutures or purse-string sutures. At the end of the procedure, we typically close the chest and we tunnel the cannula through soft tissues via separate incisions, however as needed we left the chest open. In terms of weaning ECMO we began by weaning pressor and inotropic support. We weaned down the ECMO flow (500 cpm) and volume as we continued to diuresis. We monitored vitals as we attempted to wean ECMO. We employed lung protection ventilation as we worked to wean ECMO. Weaning continued until patients are on minimal settings with stable vitals.

### Data collection

After obtaining Hartford Hospital IRB approval (HHC-2019-0189), we retrospectively reviewed prospectively collected data in our ECMO patient database and electronic health record. The primary outcome was survival to discharge. Demographics were collected to delineate age, gender, Body Mass Index (BMI), total hospital days admitted, total days on ECMO support, and ECMO type/modality. The following comorbidity data was also collected: diabetes, hypertension, coronary artery disease (CAD), history of myocardial infarction (MI), history of prior CABG, chronic obstructive pulmonary disease (COPD), asthma, congestive heart failure (CHF), peripheral vascular disease (PVD), renal disease, history of dialysis, autoimmune disease, malignancy, hyperlipidemia, pre-operative ejection fraction (EF), and pulmonary hypertension. Pre-operative ejection fraction was defined as the EF before undergoing any procedure, including catheterization laboratory procedures.

Cases were classified as elective, urgent and emergent using standard Society of Thoracic Surgery definitions. Elective cases were classified as the patient’s cardiac function has been stable for days or week prior to the operation, therefore, the procedure could be deferred without increased risk of compromised cardiac outcome. Urgent cases were classified as procedures required during the same hospitalization. These patients had to remain in the hospital until the surgery could take place, but the patient is able to wait for surgery until the next available operating room scheduled time. Emergent cases were classified as requiring emergency operations. These patients would have had ongoing, refractory, unrelenting cardiac compromise, with or without hemodynamic instability, which not responsive to any form of therapy. An emergency operation is one in which there should be no delay in providing operative intervention.

Perioperative and postoperative ECMO data was analyzed with respect to blood product use, overall volume status, and whether we placed ECMO at an outside hospital (ECMO-on-the-go) or in our institution. Postoperative complications included renal failure, sepsis, intracranial hemorrhage/anoxic brain injury, deep venous thromboembolism/pulmonary embolus (DVT/PE), ischemic limb, and cardiac reoperation. Renal failure was defined according to the Second International Consensus Conference of the Acute Dialysis Quality Initiative Group. Sepsis was defined as systemic inflammatory response syndrome (SIRS) with any known source. Anoxic brain injury in our study was defined as any imagining findings that demonstrated anoxic brain injury and confirmed by supporting documentation in the patient’s chart by a physician. The patients were divided into two groups: those patients that survived to discharge and those that expired after ECMO postoperatively.

### Statistical analysis

Continuous data is presented as a mean ± standard error or deviation if normally distributed, or as a median and interquartile range (25th, 75th) if non-normally distributed. Continuous variables were assessed with a Student’s t-test when normally distributed and Wilcoxon signed rank-sum test when data is skewed. Fisher’s exact test was used to assess categorical variables. Mann-*U* Whitney test was used to assess rank sums of data not normally distributed. Significant differences were denoted by a *p* value of ≤ 0.05 in all statistical analyses with a 95% confidence interval. We performed a descriptive analysis focusing on clinical outcomes assessed. Statistical analysis was carried out with IBM™ SPSS Statistics for Windows/Mac, version 26 (IBM Corporation, Armonk, NY 2019) and Graphpad Prism for Windows/Mac, version 8.31 (GraphPad Software, La Jolla California USA).

## Results

A total of 60 patients were included in this study (Table 1). In-hospital mortality was 60.7% (*n* = 37) with 23 patients surviving to discharge. 52 of 60 (86.6%) patients had cardiogenic shock requiring V-A ECMO (Table 2), 7 of 60 (11.6%) had respiratory failure requiring V-V ECMO and 1 (1.6%) patient had hemorrhagic shock and was placed on V-A ECMO (Table 2). The one patient who experienced hemorrhagic shock had a prolonged hospital course, ultimately requiring ECMO cannulation after sternal wound dehiscence led to profound bleeding. 2 patients were placed on V-A-V ECMO after being initially placed on V-V ECMO and were included in the V-V cohort, as outlined above.

**Table 1:** Patient demographics

Demographics	Non-survivors (N total = 37)	Survivors (N total = 23)	*p*
Age (y) (Mean ± STD)	66 ± 1.5	52 ± 3.3	< 0.001
Male	22	17	0.59
Female	14	7	0.59
BMI (kg/m^2^) (Mean ± STD)	34 ± 1.4	31 ± 1.4	0.16
*Past medical history*			
Diabetes	8	8	0.38
Hypertension	23	13	0.30
Coronary artery disease	19	6	0.037
Myocardial infarction	6	2	0.46
Pre-op coronary artery bypass graft	7	1	0.12
Chronic obstructive pulmonary disease	5	0	0.077
Asthma	5	3	1.0
Congestive heart failure	14	8	0.78
Peripheral vascular disease	2	0	0.51
Renal disease	7	6	0.75
Dialysis	3	2	1.00
Autoimmune disease	6	0	0.072
Malignancy	5	0	0.0768
Hyperlipidemia	18	9	0.43
Pulmonary hypertension	3	1	0.64
Smoker	16	7	0.41
History of cardiac surgery	8	1	0.13

*STD* standard deviation, *OHT* open heart transplant

**Table 2:** ECMO indications, timing of ECMO placement, and type of ECMO

Indications for ECMO	Non-survivors (N total = 37)	Survivors (N total = 23)	p
Cardiogenic shock	33	19	0.25
Pulmonary insufficiency	3	4	0.42
Other (hemorrhagic shock)	1	0	1.00
*ECMO type*			
VA	34	19	< 0.00001
VV	2	3	0.38
VAV	1	1	1.00
*Timing of ECMO placement*			
Pre-operatively (catheterization laboratory)	2	5	0.09
Intra-operative (operating room)	29	14	0.24
Post-operatively (ICU post-operative)	6	4	1.00

*VA* venoarterial, *VV* venovenous, *VAV* Veno-arterio-venous

43 of 60 (71.6%) were placed on ECMO in the OR, 7 (11.6%) were placed on peripheral ECMO pre-operatively in the catheterization laboratory and 10 (16.6%) were placed on peripheral or central ECMO post-operatively in the intensive care unit (ICU).

14 of 43 (32%) patients, survived having ECMO placed in the operating room. 5 of 7 (71.1%) patients survived pre-operative ECMO placement in the catheterization laboratory. 4 of 10 (40%) patients survived ECMO placement post-operatively in the ICU (Table 2).

33 of the 43 patients who had ECMO placed in the OR were centrally cannulated. 5 of 10 patients who had ECMO placed in the ICU were cannulated centrally because their chests were already open. All catheterization lab patients had peripheral cannulation. Of the 43 patients who had ECMO placed in the OR, 35 received V-A ECMO. 4 of 7 patients who had ECMO placed in the catheterization laboratory required V-A ECMO. 9 of 10 patients placed on ECMO in the ICU were placed on V-A ECMO. There was no significant difference between survivors and non-survivors in time of ECMO implantation, pre-operatively (catheterization laboratory), intra-operatively or post-operatively (in the ICU). All patients who had pre-operative ECMO placed in the catherization then proceeded directly to the OR.

Overall duration of ECMO support in all patients was a median of 5 days (2–8 days). Between survivors and non-survivors, there was no difference in the number of days patients were on ECMO (survivors 4.8 days vs non-survivors 5.7 days, *p* = *0.33*) (Table 3). However, we did find that fluid balance was significantly different between survivors and non-survivors. Survivors achieved a net negative fluid balance while on ECMO (− 3.5 L total in survivors vs + 3.4 L total in non-survivors, *p* = 0.01) (Table 3).

**Table 3:** ECMO outcomes

Outcome	Non-survivors (N total = 37)	Survivors (n = N Total = 23)	*p*
Total hospital LOS (days)	20 ± 1.5	35 ± 4.0	0.03
ECMO support (days)	5.7 ± 0.73	4.8 ± 0.53	0.33
Blood product use while on ECMO (mean units of PRBCs ± STD)	25 ± 2.3	13 ± 2.3	0.02
Bleeding events	19 (51.3%)	8 (34.7%)	0.28
Net volume status (L mean ± STD)	3.4 ± 1.6	(−) 3.5 ± 1.5	0.01
Inotrope/vasopressor use	20 (54.0%	7 (30.4%)	0.064
eCPR	15 (83.3%)	3 (16.6%)	0.02
ECMO on the go	4 (44.4%)	5 (55.5%)	0.29
OHT as salvage procedure	0	4	0.018

*LOS* length of stay, *STD* standard deviation, *OHT* open heart transplant

Of the 23 patients who survived to discharge 2 patients were discharged with an LVAD, with one of those patients ultimately receiving an Orthotropic Heart Transplant (OHT). Of the other 21 patients who survived, 6 underwent OHT, 15 recovered sufficiently for decannulation and discharge. All patients who survived were discharged either to home or a rehabilitation facility, with every patient eventually being transitioned home.

9 of 60 (15%) patients underwent ECMO-on-the-go. 5 of these 9 (55.5%) patients survived. However, this was not statistically different (p = 0.29).

### Demographics

Survivors were found to be younger (52 years vs 66 years, *p* < 0.001) (Table 1). The average age of all patients was 60 ± 1.79 years old. 19 of 37 (51.3%) non-survivors had a past medical history of CAD. This was the only past medical history analyzed that was found to be significantly more prevalent in non-survivors than survivors (p = 0.037). Overall, our patient’s mean preoperative EF was 42%, this was not significantly different between survivors and non-survivors (p = 0.30).

22 of 60 (36.6%) cases were elective, 10 of 60 (16.6%) cases were emergent, and 28 of 60 (45%) cases were urgent (Table 4). There was no statistical difference between survivors and non-survivors based on operation classification (p > 0.05). In addition, ECMO cardiopulmonary resuscitation was initiated either in the operative theater or post-operatively in the ICU. ECMO cardiopulmonary resuscitation (eCPR) had a high incidence of mortality. 12 of 15 (80%) ECMO patients who had eCPR did not survive (Table 3).

**Table 4:** Operation classification

Operation classification	Non-Survivors (N total = 37)	Survivors (N total = 23)	*p*
Elective	16	6	0.27
Emergent	7	3	0.73
Urgent	14	14	0.19

12 of 60 (20%) operative cases were isolated CABGs. Non-survivors (n = 10) had a mean of 2.86 vessel disease repaired whereas survivors (n = 2) had a mean of 3.75 vessel disease repaired. 17 of 60 (28.3%) patients underwent isolated valve repair. 6 of 60 (10%) patients underwent CABG and valve repair. 7 of 60 (11.7%) patients underwent aortic root repair. 4 of 60 (6.6%) patients had a LVAD placement. 2 of 60 (3.3%) patients underwent descending aortic repair. 8 of 60 (13.3%) patients underwent less commonly performed open heart procedures. 4 of 60 (6.6%) patients underwent OHT as their primary surgery (Table 5).

**Table 5:** Primary surgery

Primary surgery	Non-survivors (N total = 37)	Survivors (n total = 23)	*p*
CABG	10	2	0.16
Valve repair	10	7	0.77
CABG + valve repair	4	2	1.00
OHT	2	2	0.04
Aortic root repair	5	2	0.69
LVAD placement	3	1	1.00
Descending aortic repair	0	2	0.14
Less commonly performed procedures	2	6	0.04

*CABG* coronary artery bypass surgery, *OHT* open heart surgery, *LVAD* left ventricular assist device

However, in total 8 of 60 (13.3%) patients underwent OHT. 4 patients had OHT as their primary procedure and required ECMO to be brought out of the OR. 2 of the patients who had OHT performed as a primary procedure did not survive. Primary OHT was the only heart surgical procedure found to be significantly different between survivors and non-survivors (p = 0.04). 4 patients underwent OHT after being placed on ECMO as a salvage for their original cardiac surgical procedure. These 4 patients already had a primary open-heart surgery and OHT was attempted as a salvage procedure.

### ECMO secondary outcomes and adverse events

19 of 37 non-survivors (51.3%) had a bleeding event while on ECMO. 5 of these 19 patients needed re-operation of their chest. 8 of 23 (34.7%) survivors had a bleeding event on ECMO. 2 of these 8 patients needed re-operation of their chest for bleeding. This was not significantly different between survivors and non-survivors, but trending towards increased bleeding events in non-survivors (p = 0.28). Survivors needed a mean 13 units of blood, while non-survivors needed a mean of 25 units of blood, this was found to be significantly different (*p* = 0.02).

14 of 37 (37.8%) non-survivors required re-operation, which was significantly more than survivors (p = 0.04). 3 of 23 (13.0%) survivors required re-operation, 2 for bleeding and 1 for sternal wound dehiscence. 20 (54.0%) non-survivors and 5 (21.7%) survivors developed renal failure requiring renal replacement therapy without significant difference between these groups (p = 0.16). 18 (48%) non-survivors and 13 (56.5%) survivors developed sepsis, again, not statistically different (p = 0.6). 8 (21.6%) non-survivors and 1 (4.34%) survivor developed intracerebral hemorrhage/anoxic brain injury which was not significantly different (p = 0.13). 1 (2.7%) non-survivor and 2 (8.6%) survivors developed DVT/PE, which was not significantly different (p = 0.56). 4 Patients (3 survivors), had clotting in their ECMO circuit, of these 2 patients were not anticoagulated with heparin and occurred at a mean of 10 days. Of note, one patient had thrombosis of the circuit in the OR and did not survive.

## Discussion

ECMO is a valuable therapeutic option for postcardiotomy, particularly when the underlying cause is thought to be reversible [4–7, 11–13]. This series demonstrates a heterogenous range of cardiac procedures with a similar duration of ECMO support. Determining risk profile in those patients that survive ECMO demonstrates important findings that may suggest means to improve hospital management.

Our paper focuses on increasing survival in patients who do not survive cardiac surgery. We believe ECMO use helps stabilize these patients for eventual discharge. In our patient cohort, in-hospital mortality was 60%. The literature on ECMO placement following cardiac surgery has similar mortality rates of 64–67% representing the overall tenuous underlying pathophysiology in these patients [14, 17]. Without ECMO use, we believe the 40% of survivors in our study would have died without mechanical circulatory support [15, 17].

Our observed mean duration of ECMO support was 5 days, similar to that reported of 5.4–7.5 days, without significant difference for survivors and non-survivors [14, 15, 17]. Therefore, we suggest transparent discussion with families and patients regarding potential outcomes and allowing up to 8 days on ECMO before making vital decisions. One marker that stood out as a predictor of survival in our study is achieving overall net negative fluid balance (− 3.5 L vs + 3.4 L, *p* = 0.01). This suggests restoration of sustainable hemodynamics and resolution of ongoing capillary leak. This may be a good indicator for potential survivability, although it cannot unfortunately be predicted when first implanting ECMO. A strong focus on diuresis is a critical component to ECMO patient survival.

There were similar occurrences of bleeding events between patients that survived and those that did not survive, with a lower number of units of blood needed for resuscitation, for those patients that did survive (mean units of PRBCs for survivors was 2.7 units a day, compared to 4.7 units of PRBCs a day in non-survivors, *p* = 0.02). Our patients required similar rates of red blood cell transfusions compared to prior studies [15–17]. Directed resuscitation with appropriate blood product use improves survival, but to our knowledge there have been no other studies measuring volume status and blood product usage [3, 10, 18]. Our study unmasks this potential link, therefore we suggest, appropriate diuresis and directed resuscitation suggest an important therapy to bridge a distressed myocardium and may aid the efforts of ECMO support.

Survival for V-A ECMO was 35% compared to 60% in V-V ECMO. Even though this was found insignificant, it is unsurprising. Patients with two rather than one organ failing are more likely to have poor outcomes.

Cardiac reoperations were associated with worse survival outcomes while patients were on ECMO. Kidney failure, DVT/PE, ischemic limb, sepsis and intracerebral hemorrhage/anoxic brain injury had no significant mortality detriment, however there is a trend toward worse survival, with these mal-occurrences. Overall, only 3 patients had a DVT/PE. One thought for our overall low DVT/PE rate is our patients placed on VA ECMO either had prophylactic superficial femoral artery catheter or had regional SO^2^ monitors on both feet showing good perfusion in the ipsilateral foot of the ECMO arterial side. We believe that in a larger sample size these values would become significant because we know they are significant in heart surgery patients who do not need ECMO.

We believe ECMO is a method for cardiopulmonary support to allow the body time to heal and to support end organ perfusion, as a bridge to recovery or to a more permanent treatment solution such as transplant or LVAD. In our patients, a bridge-to-recovery approach was anticipated in the vast majority of the patients despite this cohort showing varying degrees of chronic heart failure pre-operatively. It has been shown that the potential for recovery may be limited in patients with underlying chronic heart failure and combined with advanced age, ECMO may be a futile intervention in the end. In resuscitated patients, a bridge-to-decision strategy for ECMO’s use may be employed to help manage subsequent therapy such as LVAD surgery, implantable cardioverter defibrillator (ICD) implantation, and OHT. If the patient leaves the hospital with their own heart, it is important to avoid LV distention. Consequently, on ECMO support it is our recommendation to ensure adequate off-loading of the left ventricle (usually with an LV vent or Impella) and to minimize inopressor use to reduce myocardial oxygen demand and allow for myocardial recovery. It is unclear if this off-loading should be done for all patients preemptively when placed on ECMO support or if only after cardiac ECHO evaluation suggests that the LV is distended. We generally employee off-loading when it is indicated by ECHO, but at this point we do not have any standard policy. Going forward this would be an interesting topic of research and standardization. In our 23 patients who survived two patients left with LVADs and 6 patients left with OHTs, 8 patients had some kind of assistance or another heart placed. More study is needed in this regard to help streamline the post-operative resuscitative course of patients who require post cardiotomy mechanical support.

If a patient’s heart is failing there are options, one of which is ECMO. While these patients have drastically worse outcome than if they did not need ECMO, we think that it may allow us to salvage patients who are unsavable.

## Conclusion

ECMO is a viable rescue strategy for cardiac surgery patients with a 40% survival to discharge rate. Careful attention to volume management and blood transfusion are important markers for potential survival.

## Data Availability

The datasets generated and/or analysed during the current study are not publicly available due to patient personal information included in the primary data set, but are available from the corresponding author on reasonable request.

## Abbreviations

ECMO: Extracorporeal membrane oxygenation
CABG: Coronary artery bypass grafting
LVAD: Left ventricular assistant device
VA: Venous-arterial
VV: Venous-venous
VAV: Veno-arterio-venous
IABP: Intra-aortic ballon pump
CAD: Coronary artery disease
MI: Myocardial infarction
COPD: Chronic obstructive pulmonary disease
CHF: Congestive heart failure
PVD: Peripheral vascular disease
EF: Ejection fraction
DVT/PE: Deep venous thromboembolism/pulmonary embolus
ICU: Intensive care unit
OHT: Open heart transplant
eCPR: ECMO cardiopulomary resuscitation
PRBCs: Packed red blood cells
ICD: Implantable cardioverter defibrillator
